# Endoscopic retrograde cholangiopancreatography combined with peroral choledochoscope for the treatment of complete bile duct rupture

**DOI:** 10.1055/a-2512-4565

**Published:** 2025-02-18

**Authors:** Qingsong Wu, Ming Zhang, Donghai Zhuang, Baochang Shi, Jinglong Guo, Yannan Yin, Kai Zhang

**Affiliations:** 1 582805 Department of Hepatobiliary Surgery, Shandong Provincial Third Hospital, Jinan, China; 2 Department of Hepatobiliary Surgery, Shandong Provincial Third Hospital, Jinan, China

Iatrogenic bile duct injury is one of the serious complications of laparoscopic cholecystectomy 1 2 . Here, we present a patient with massive bile leakage due to complete bile duct rupture after the procedure. A 69-year-old man was admitted to the hospital due to skin and sclera jaundice with a fever for half a month. He underwent laparoscopic cholecystectomy in another hospital 20 days ago. The abdominal drainage tube drained about 800 ml of bile daily. Magnetic resonance cholangiopancreatography (MRCP) showed localized ascites and discontinuity of the common bile duct (CBD) ( Fig. 1 ).

**Fig. 1:**
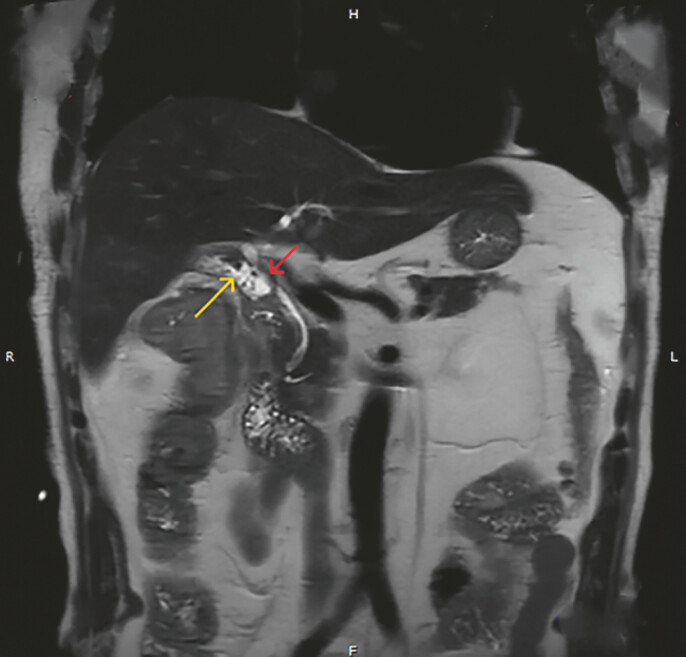
Magnetic resonance cholangiopancreatography showed localized ascites (yellow arrow) and discontinuity of the common bile duct (CBD) (red arrow).

Endoscopic retrograde cholangiopancreatography (ERCP) showed contrast agent extravasation into the peritoneal cavity and rupture of the upper CBD ( Fig. 2 ). We then explored the CBD through the peroral choledochoscope and found that the upper part of the CBD was completely ruptured. The abdominal drainage tube and Hem-o-lok clip were seen through the peroral choledochoscope. After repeated attempts, combined with X-ray localization, we successfully inserted guidewires into the left and right hepatic ducts ( Video 1 ). The common hepatic duct, left hepatic duct, and right hepatic duct were identified through the peroral choledochoscope ( Fig. 3 ). A plastic stent (8.5 Fr, 12 cm) was placed in the right hepatic duct, followed by a fully covered metal stent (10 mm, 5 cm) in the CBD. Finally, a nasobiliary duct was placed in the left hepatic duct through the metal stent lumen ( Fig. 4 ). Bile in abdominal drainage decreased rapidly to disappear. On the 10 ^th^ day after the intervention, nasal cholangiography showed no obvious bile leakage ( Fig. 5 ). On the 24 ^th^ day, it showed the stent was unobstructed without stenosis.

**Fig. 2:**
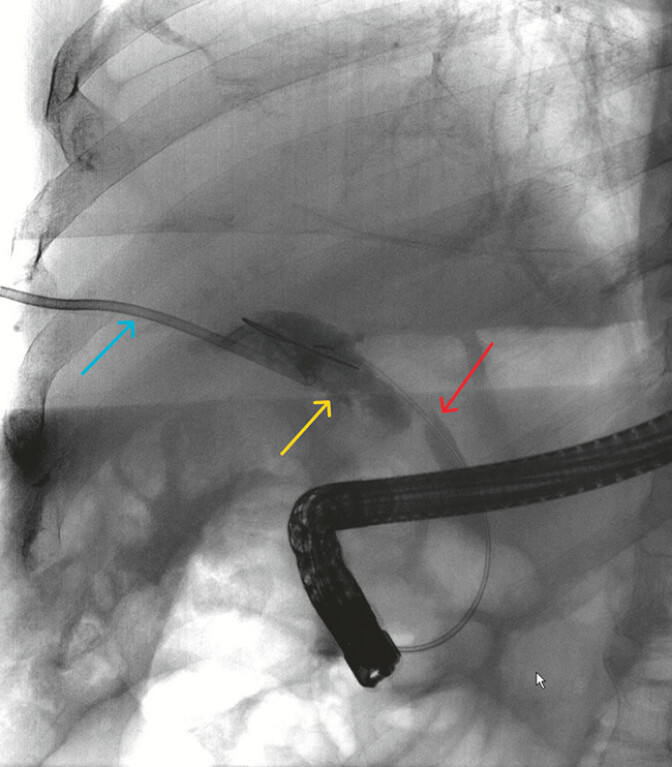
Endoscopic retrograde cholangiopancreatography showed contrast agent extravasation into the peritoneal cavity (yellow arrow) and rupture of the upper CBD (red arrow). The blue arrow was the abdominal puncture drainage tube.

**Fig. 3:**
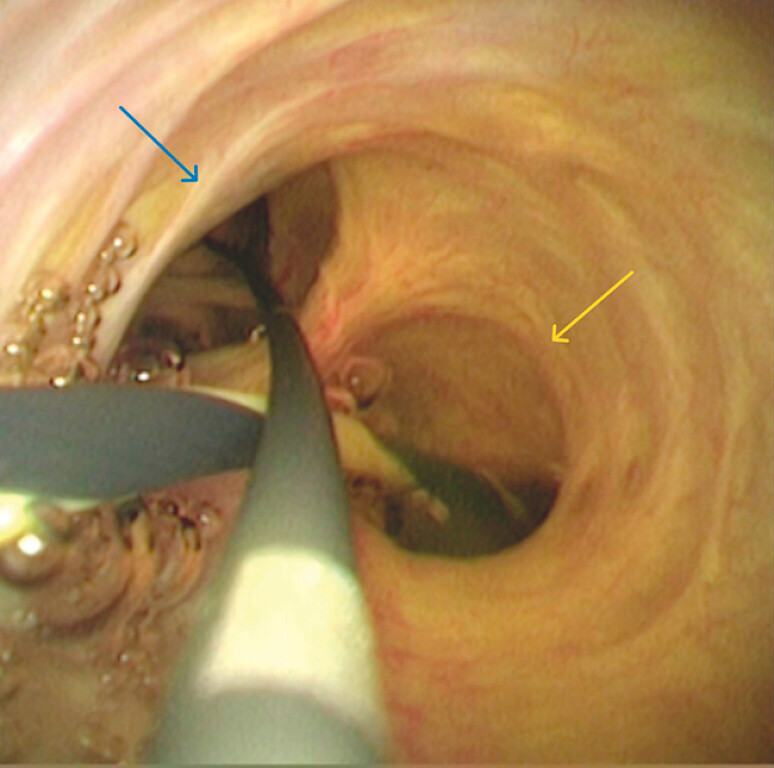
The common hepatic duct, left hepatic duct (yellow arrow), and right hepatic duct (blue arrow) were identified under peroral choledochoscope.

**Fig. 4:**
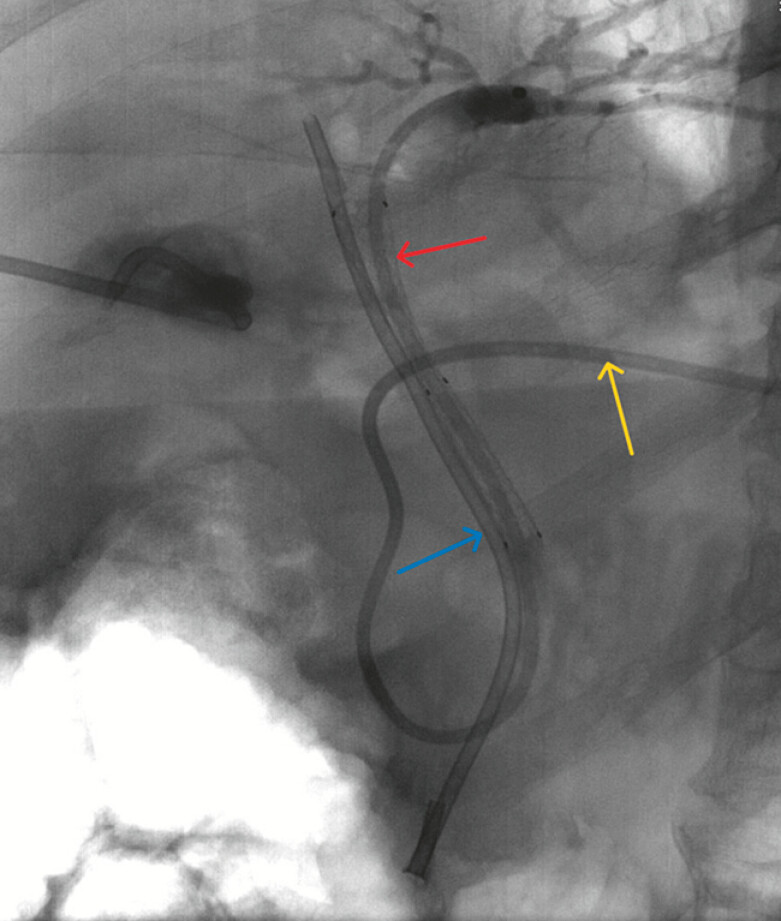
A plastic stent (blue arrow) was placed in the right hepatic duct, followed by a fully covered metal stent (red arrow) in the CBD. A nasobiliary duct (yellow arrow) was placed in the left hepatic duct through the metal stent lumen.

**Fig. 5:**
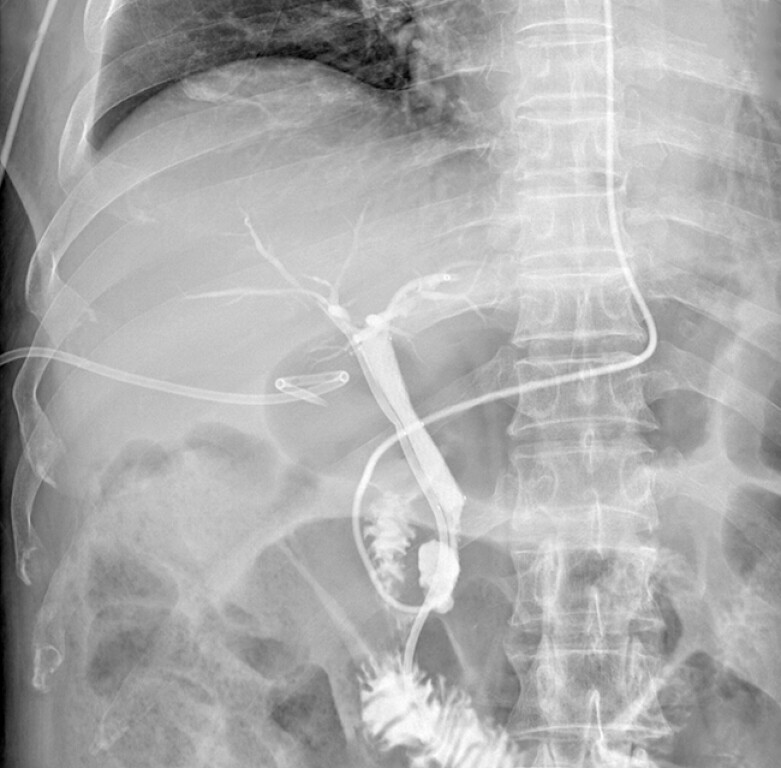
On the 10 ^th^ day after the intervention, nasal cholangiography showed no obvious bile leakage.

Endoscopic retrograde cholangiopancreatography combined with peroral choledochoscope for the treatment of complete bile duct rupture.Video 1

Cases with a partial defect or rupture of the bile duct after laparoscopic cholecystectomy usually require secondary surgery 3 . ERCP combined with peroral choledochoscope to bridge the ruptured bile duct has created a new, alternative minimally invasive treatment approach. However, long-term effects such as biliary stenosis require longer follow-up and more cases to provide experience.

Endoscopy_UCTN_Code_CPL_1AM_2AZ

## Notes

Endoscopy E-Videos https://eref.thieme.de/e-videos
*E-Videos* is an open access online section of the journal *Endoscopy* , reporting on interesting cases and new techniques in gastroenterological endoscopy. All papers include a high-quality video and are published with a Creative Commons CC-BY license. Endoscopy E-Videos qualify for HINARI discounts and waivers and eligibility is automatically checked during the submission process. We grant 100% waivers to articles whose corresponding authors are based in Group A countries and 50% waivers to those who are based in Group B countries as classified by Research4Life (see: https://www.research4life.org/access/eligibility/ ). This section has its own submission website at https://mc.manuscriptcentral.com/e-videos .
